# La recherche de la surexpression de la protéine et l'amplification du gène HER2 dans le cancer de l'estomac par immunohistochimie et Hybridation in Situ: expérience du CHU HASSAN II de Fès

**Published:** 2012-12-17

**Authors:** Hinde El Fatemi, Nawal Hammas, Karima Idrissi, Nawfel Mellas, Amal Bennani, Afaf Amarti, Anne Cayre, Frederique Penault-llorca, Omar Mesbahi

**Affiliations:** 1 Laboratoire d'anatomie et cytologie pathologique, CHU HASSAN II, FES, Maroc; 2 Laboratoire d'anatomie et cytologie pathologique, Jean Perrin, Clermont Ferrand, France; 3 Service d'oncologie médicale, CHU HASSAN II, FES, Maroc

**Keywords:** Protéine, amplification, HER2, cancer, immunohistochimie, hybridation in situ, Protein, amplification, HER2, cancer, immunohistochemistry, in situ hybridation

## Abstract

La surexpression de l'Her2 a été détectée dans plusieurs cancers et a été particulièrement étudiée dans le cancer du sein. Elle est décrite dans 10 à 30% des adénocarcinomes gastriques. Un statut HER2 positif est un facteur de mauvais pronostic et un facteur prédictif de la réponse à l'herceptine (trastuzumab). Cet article présente les résultats d'une étude préliminaire portant sur 31 cas de tumeurs gastriques, et dont le but est d’évaluer la surexpression de l’ HER2 dans les adénocarcinomes gastriques avancés tout en comparant nos résultats avec ceux de la littérature. Le taux des cas surexprimant l'Her2 dans notre étude (35.5%) est proche de celui noté par Aoyaji et al (34%), mais il est supérieur à celui noté dans la plupart des séries de la littérature notamment une vaste étude baptisée TOGA. La poursuite de cette étude par un échantillon plus large est nécessaire afin de mieux comprendre les particularités de ce cancer dans notre contexte.

## Aux éditeurs du Journal Panafricain de Médecine

L'amplification et la surexpression de l'Her2 (human epidermal growth factor receptor 2) ont été détectées dans plusieurs cancers et ont été particulièrement étudiées dans le cancer du sein [1]. Elles sont décrites dans 10 à 30% des adénocarcinomes gastriques [2, 3]. Un statut HER2 positif est un facteur de mauvais pronostic et est associé à une maladie plus agressive [4]. C'est aussi un facteur prédictif de la réponse à l'herceptine (trastuzumab), anticorps monoclonal humanisé dirigé contre l'HER 2, utilisé depuis plusieurs années dans les cancers du sein surexprimant l'HER2 en situation métastatique et adjuvante, et qui est indiqué dans les adénocarcinomes de l'estomac, du bas oesophage et de la jonction oeso-gastrique localement avancés et /ou métastatiques [1, 5]. Au Maroc, l'herceptine n'a pas encore l'AMM pour le cancer de l'estomac. Cet article est le premier travail fait au Maroc et présente les résultats d'une étude préliminaire portant sur 31 cas de cancers gastriques, et dont le but est d’évaluer la surexpression et/ ou l'amplification de la protéine et du gène HER2 dans les adénocarcinomes gastriques avancés tout en comparant nos résultats avec ceux de la littérature. Notre travail porte sur 31 cas de tumeurs gastriques colligées au laboratoire d'anatomie pathologique sur une durée de deux ans. Le diagnostic a été posé sur 22 biopsies et 09 pièces de résection chirurgicale. Le statut HER2 a été déterminé par techniques immunohistochimique et par hybridation in situ fluorescente (FISH), réalisées sur prélèvement fixé dans le formol et inclus en paraffine. L'interprétation de l'immunohistochimie s'est basée sur l’évaluation de l'intensité du marquage et le pourcentage de cellules tumorales marquées. Un seuil de 10% de cellules tumorales marquées a été retenu pour les pièces chirurgicales et 5% pour les biopsies vu l'hétérogénéité de l'immunomarquage par l'HER2 dans le cancer de l'estomac. Les scores 0 et 1 sont considérés comme négatifs; le score 3 comme positif et le score 2 comme équivoque. Dans cette dernière situation, la recherche de l'amplification de l'HER2 a été réalisée par FISH. Cette étude n'inclus par la survie des malades, vu la non autorisation de mise en marché du Trastuzumab pour les cancers gastriques au Maroc. L’âge de nos patients est compris entre 21 et 84 ans avec un âge moyen de 52 ans. Une nette prédominance masculine est observée avec 24 hommes et 7 femmes, soit un sex ratio de 3,2. La localisation antro-fundique est retrouvée dans 29 cas, alors que la localisation oeso-gastrique est notée dans uniquement deux cas. Tous les malades se sont présentés à un stade avancé avec des métastases (au moins pT3N +). Notre étude a porté sur 24 cas d'adénocarcinomes et 7 cas de carcinomes à cellules indépendantes. A l’étude immunohistochimique, la recherche de la surexpression de l'Her2 a montré que 8 (25,8%) cas étaient de score 0, 8(25,8%) cas de score 1, 5 (16,1%) cas de score 2 et 10(32,3%) cas de score 3. Un complément par FISH a été réalisé seulement sur les cas de score 2 dont un a été amplifié. Le taux de surexpression est ainsi de 35.5% dans notre série. Les résultats épidémiologiques dans notre série concordent avec ceux de la littérature. En effet, ce cancer est deux fois plus fréquent chez les hommes que chez les femmes et il touche plus particulièrement les personnes de plus de 55 ans [6]. Par contre, des différences sont enregistrées dans notre série par rapport aux résultats de l'ASCO 2009 concernant la localisation (Tableau 1). Par ailleurs aucune différence n'a été observée pour le pTNM ni pour le stade [7].

**Tableau 1:** Comparaison des résultats de notre série avec ceux de l'American Society of Clinical Oncology (ASCO) concernant le type histologique

	ASCO 2009	notre série
Type intestinal	32.2%	77,4%
Type diffus	6.1%	22.6%

Le cancer de l'estomac avancé est grevé d'un mauvais pronostic; la durée moyenne de survie après le diagnostic est d'environ 10-11 mois avec les traitements actuellement disponibles [8]. Un diagnostic précoce de cette maladie est difficile, car la plupart des patients restent longtemps asymptomatiques. La mise en place de nouvelles thérapeutiques ciblées devient alors de plus en plus nécessaire pour améliorer le pronostic. Dans ce cadre, et en raison des effets secondaires de ces thérapeutiques, la détermination du statut HER2 est devenue indispensable pour sélectionner les patients qui vont en bénéficier. [1]. Cette surexpression est considérée comme un facteur pronostique péjoratif et elle est liée à une diminution de la survie et à un potentiel agressif de la maladie. La surexpression de l'HER2 est définie par un HER2 score 2 confirmé par une amplification du gène de l'HER2 par FISH, ou par un score 3. Pour les cas score 3, on prend en compte un marquage en « U », fort, incomplet, basolatéral ou latéral avec un seuil de 10% de cellules marquées pour les pièces opératoires et le marquage d'au moins cinq cellules adjacentes cohésives pour les biopsies. Pour l'analyse FISH, le gène de l'HER2 est considéré comme amplifié si le ratio HER2/CEP17 est supérieur ou égal à 2 (CEP: centromère du chromosome 17). Le taux des cas surexprimant l'Her2 (score 3 et score 2 avec amplification de l'Her2 par FISH) dans notre étude (35.5%) est proche de celui noté par Aoyaji et al (34%) [9], mais il est supérieur à celui noté dans la plupart des séries de la littérature notamment une vaste étude baptisée TOGA qui a montré des taux de surexpression de 22.1% des cas [1]. L'expression d'Her2 est plus fréquente dans les adénocarcinomes du cardia qui sont de type intestinal et bien différenciés [1]. Les résultats de l’étude ToGA ont montré également que l'adjonction du trastuzumab améliorait significativement la médiane de survie de près de trois mois sans effets secondaires notables [1]. Sur la base de ces résultats, la demande d'extension du champ d'application d'Herceptine a bénéficié d'une procédure d'examen accélérée par les autorités de santé européennes, ce qui a permis plus rapidement aux patients d'avoir accès à ce médicament qui prolonge la survie. L'autorisation de mise sur le marché vaut avec effet immédiat pour toute l'Union européenne (UE) ainsi que pour l'Islande, le Lichtenstein et la Norvège, Etats membres de l'EEE/de l'AELE. Suite à son homologation dans l'Union européenne, l'Herceptine devrait bientôt bénéficier d'une extension de son champ d'application dans d'autres régions du monde. Au Maroc, l'herceptine n'a pas encore d'AMM dans le cancer de l'estomac. Ce traitement est indiqué chez les patients porteurs d'une tumeur gastrique avancée ou métastatique, HER2-positive en association avec capécitabine ou 5-FU et cisplatine pour le traitement des patients avec adénocarcinome HER2 positif métastatique de l'estomac ou de la jonction oeso-gastrique qui n'ont pas reçu de traitement pour leur maladie métastatique.

## Conclusion

Vu le mauvais pronostic du cancer de l'estomac, la recherche de la surexpression de l'Her2 est devenue indispensable pour sélectionner les patients candidats à un traitement par l'Herceptine. Notre série se caractérise par un taux d'expression supérieur à celui de la plupart des séries de la littérature. La poursuite de cette étude par un échantillon plus large est nécessaire afin de mieux comprendre les particularités de ce cancer dans notre contexte.

## References

[1] Hofmann M, Stoss O, Shi D, Buttner R (2008). Assessment of a HER2 scoring system for gastric cancer: results from a validation study. Histopathology..

[2] Park Dl (2006). HER2/neu amplification is an independent prognostic factor in gastric cancer. Dig Dis Sci.

[3] Marx AH, Tharun L, Muth J, Dancau AM (2009). HER2 amplification is highly homogenous in gastric cancer. Hum Pathol..

[4] Garcia I (2003). Clinical significance of the epidermal growth factor receptor in resectable gastric cancer. Ann Surg Oncol..

[5] Bang YG (2010). Trastuzumab in combination with chemotherapy versus chemotherapy alone for treatment of HER2- positive advanced gastric or gastro-oesophaageal junction cancer (TOGA): a phase 3, open-label, randomised controlled trial. Lancet..

[6] Marx AH, Burandt EC, Choschzick M (2010). Heterogenous high-level HER-2 amplification in a small subset of colorectal cancers. Hum Pathol..

[7] Liang Z, Zeng X, Gao J, Wu S (2008). Analysis of EGFR, HER2, and TOP2A gene status and chromosomal polysomy in gastric adenocarcinoma from Chinese patients. BMC Cancer..

[8] Ohtsu A (2008). Chemotherapy for metastatic gastric cancer: past, present, and future. J Gastroenterol..

[9] Aoyagi K, Kohfuji K, Yano S (2001). Evaluation of the epidermal growth factor receptor (EGFR) and c-erbB-2 in superspreading-type and penetrating-type gastric carcinoma. Kurume Med J..

